# High-Temperature Gelation and Structural Characterisation of Commercial Yellow Pea, Faba Bean, and Mungbean Protein–Starch Systems

**DOI:** 10.3390/gels12010089

**Published:** 2026-01-19

**Authors:** Niorie Moniharapon, Minqian Zhu, Lucinda Daborn, Sushil Dhital

**Affiliations:** 1 Faculty of Science, Monash University, Clayton, Melbourne, VIC 3800, Australia; 2 Department of Chemical and Biological Engineering, Monash University, Clayton, Melbourne, VIC 3800, Australia

**Keywords:** high-temperature gelation, legume proteins, dry-fractionated starch, pasting behaviour, rheology, gel strength, plant-based food systems

## Abstract

The heating of plant proteins at high temperatures is often associated with phase separation due to the aggregation of protein fractions, resulting in weak or discontinuous gels in liquid processing systems. This study examined the high-temperature gelation behaviour of commercial yellow pea, faba bean, and mungbean protein isolates and evaluated how different levels of dry-fractionated starch substitution tailor viscosity development and final gel strength. To characterise structural changes during heating, pasting behaviour was evaluated at 95 °C and 120 °C using a high-temperature Rapid Visco Analyser, while gel strength, temperature-ramp rheology, and thermal transitions were measured using a texture analyser, rheometer, and Differential Scanning Calorimetry. At 95 °C, all systems showed controlled pasting behaviour, with yellow pea exhibiting moderate viscosity development and clear recovery during cooling, mungbean generating the highest peak viscosity, and faba bean forming the strongest elastic network and gel structure. At 120 °C, yellow pea showed reduced stability, whereas faba bean and mungbean retained higher viscosity during heating. Starch addition improved the viscosity stability and gel strength across all proteins by limiting excessive aggregation and supporting network formation. These findings clarify how protein type and starch substitution affect high-temperature gelation, supporting the development of a heat-stable, clean-label plant-based gel system.

## 1. Introduction

The gelation behaviour of legume proteins depends strongly on the protein concentration and on the relative proportions of vicilin- and legumin-type globulins, which largely govern network formation in commercial protein isolates. Residual non-protein components and the processing history associated with industrial extraction may exert a secondary, modulatory influence on hydration, aggregation, and gel development [1,2]. Studies on mungbean, lentil, and pea show that differences in globulin composition influence the concentration needed to form an elastic network and the temperature at which gelation begins, defined by the point where G′ exceeds G″ [3,4,5]. Similar effects are reported for lupin, where variation in vicilin–legumin ratios leads to distinct gelation temperatures and network development pathways [6]. Across these legumes, increasing protein concentration accelerates the solid–gel transition because unfolding and aggregation occur more rapidly. However, at higher temperatures, particularly under shear, these proteins tend to aggregate excessively and undergo phase separation, resulting in minimal final viscosity and limiting their use in stirred high-temperature systems [3,6,7,8].

To address the limitations of protein-only systems, several studies have examined protein and starch mixtures, although the starch used usually does not originate from the same legume. Cassava and maize starch have been shown to improve network structure in faba or lupin systems [9] and other mixed formulations, such as the pea protein with carrageenan and starch blends or chickpea and faba composites, also demonstrate that starch can influence gel behaviour [10,11]. However, because these systems combine ingredients from different botanical sources, they do not necessarily capture intrinsic legume-specific interactions. In such mixed formulations, compatibility is governed primarily by molecular architecture and surface chemistry rather than their botanical origin [12]. Differences in starch granule morphology, chain length distribution, and surface charge, together with protein conformation and hydration behaviour, determine the extent of phase association or segregation during heating. This recognition has motivated a growing interest in endogenous starch systems, where protein and starch co-exist within the native legume matrix, providing a more representative framework for probing intrinsic protein–starch interactions [8,13].

Recent studies pairing legume proteins with their own starch fractions offer a clearer view of intrinsic protein–starch interactions. In faba bean systems where the protein forms the continuous phase, adding starch reduces large-deformation strength because swollen granules act as inactive fillers, while small-deformation stiffness increases as starch effectively concentrates the protein [14,15]. When starch becomes the dominant phase, gel strength and elasticity increase, although higher protein levels compete for water, delay granule swelling, and produce denser but weaker, more viscous networks [16,17]. In pea systems, the protein can instead inhibit starch-driven gelation; the pea protein isolate forms a coating around granules, suppresses swelling and amylose release, and disrupts molecular aggregation, resulting in softer, less cohesive gels [18]. Yet these studies were all conducted below 100 °C and in single-legume models, leaving high-temperature behaviour and cross-legume comparisons unclear.

A recent attempt to characterise high-temperature gelation in legume systems was made by Devkota et al., who examined how vicilin- and legumin-rich lab-extracted lupin proteins behave with maize starch at 95–120 °C. Their results showed that high temperatures accelerate vicilin denaturation and weaken gel formation, whereas legumin-rich systems form stronger, more stable networks, and waxy maize starch further increased viscosity and gel firmness [19]. Complementary evidence from extrusion studies also highlights strong temperature and shear sensitivity, where starch increases the expansion and water-holding but promotes stronger aggregation in faba systems [20,21,22,23]. Although this work established an important reference for high-temperature protein–starch behaviour, it focused only on lupin and relied on non-endogenous starch, leaving open how starch substitution shapes the viscosity and structural transitions across other legumes.

Addressing this gap is essential for understanding how heat-resistant gels can be formulated for plant-based applications. This study investigates the high-temperature pasting and gelation behaviour of faba bean, yellow pea, and mungbean protein systems with varying levels of dry-fractionated starch fraction substitution. By integrating high-temperature pasting with rheology and gel strength measurements, the study examines how the protein type, starch level, and processing temperature regulate viscosity development, gel network behaviour, and structural stability through protein–starch interactions.

## 2. Results and Discussion

### 2.1. Pasting Behaviour

**Figure 1:**
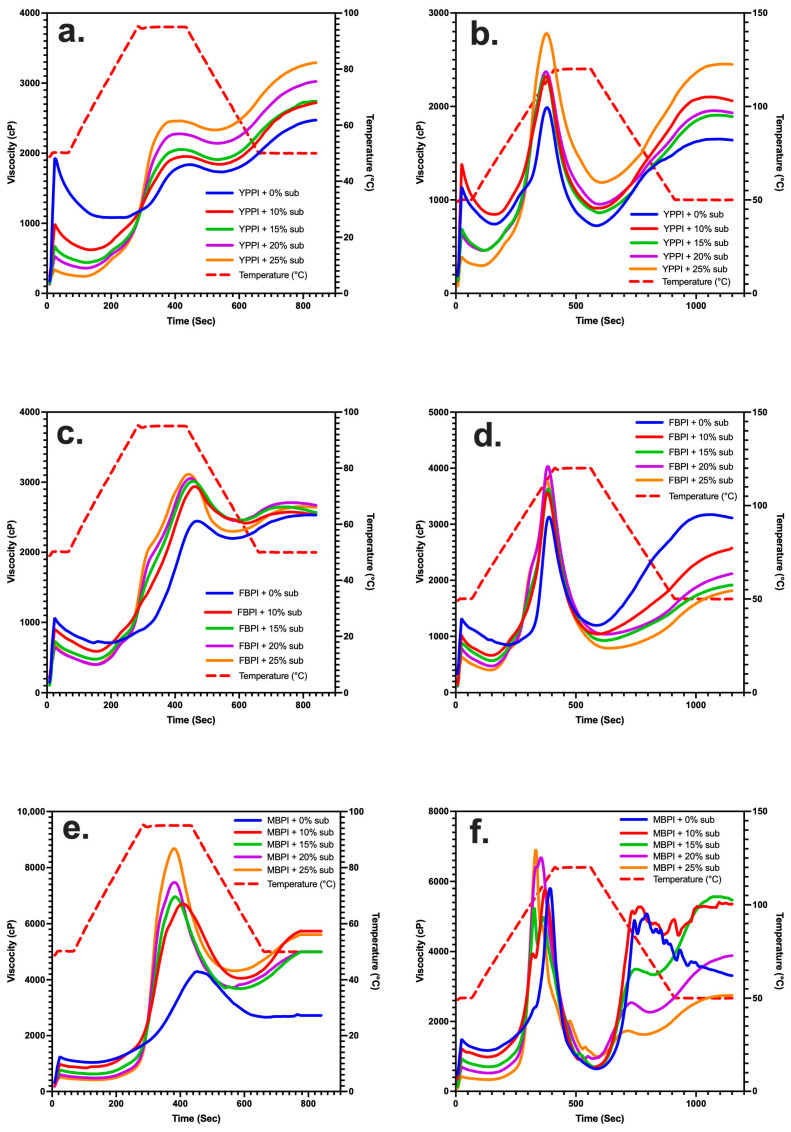
RVA pasting profiles of yellow pea (**a**,**b**), faba bean (**c**,**d**), and mungbean (**e**,**f**) protein isolates with 0–25% (*w*/*w*) dry-fractionated starch substitution at 95 °C and 120 °C; solid lines represent viscosity development and dashed lines represent the programmed temperature profile.

The Rapid Visco Analyser (RVA) provides a continuous record of viscosity development during controlled heating and cooling, allowing for the assessment of peak, trough, breakdown, final viscosity, and setback (Figure 1 and Table 1, Table 2 and Table 3) [24]. In this study, the RVA was used at both 95 °C and 120 °C to compare how starch substitution influences the pasting behaviour of the three legume protein systems, and to examine how moderate versus high-temperature conditions alter their capacity to develop and maintain viscosity.

The RVA profiles of yellow pea systems displayed a clear vicilin-driven pasting pattern, characterised by moderate viscosity development and limited thermal stability [25]. At 95 °C, yellow pea systems exhibited a more moderate and controlled pasting behaviour compared with high-temperature processing (Figure 1a). All formulations showed a gradual increase in viscosity during heating, followed by a clear peak and a subsequent decrease during holding, characteristic of starch swelling and partial granule disruption under moderate thermal conditions. Protein-only systems displayed lower initial and peak viscosities, while starch substitution progressively increased the viscosity development, with peak viscosity rising from 1814 to 2461.5 cP. This reflects the ability of dry-fractionated pea starch granules to swell and compete for water during heating [26,27]. This agrees with earlier observations that native legume starches retain granule integrity under moderate heating and contribute substantially to viscosity development when water availability is adequate [28,29]. Despite this, the increment from initial viscosity to peak remained relatively small in the yellow pea system compared with faba bean and mungbean. This indicates a gradual and constrained viscosity build-up during heating, rather than a pronounced swelling-driven response.

During cooling, viscosity increased across all formulations, resulting in final viscosity values exceeding the corresponding peaks. This trend is consistent with starch reassociation and retrogradation during cooling, as reported in starch-containing systems, and suggests that starch addition primarily amplified the viscosity magnitude without changing the overall RVA pasting profile. Similar cooling-driven viscosity increases have been reported for pea protein-containing systems and for native pea starch, where viscosity rises after holding due to starch molecule reassociation during cooling [28,30,31,32].

At 120 °C, these weaknesses became more pronounced (Figure 1b). Although peak viscosity still increased with starch (1990 to 2781.5 cP), breakdown values remained high across all substitution levels, and trough viscosity dropped sharply, confirming that yellow pea systems cannot maintain granule integrity under severe heating. High temperatures accelerate vicilin dissociation, reducing its capacity to stabilise swollen granules or form a cohesive viscoelastic matrix [33]. Similar high-temperature behaviour has been reported in previous studies, where pea proteins exhibited lower denaturation enthalpy and weaker aggregation tendencies compared with legumin-rich species such as faba bean [3,34].

**Figure 2:**
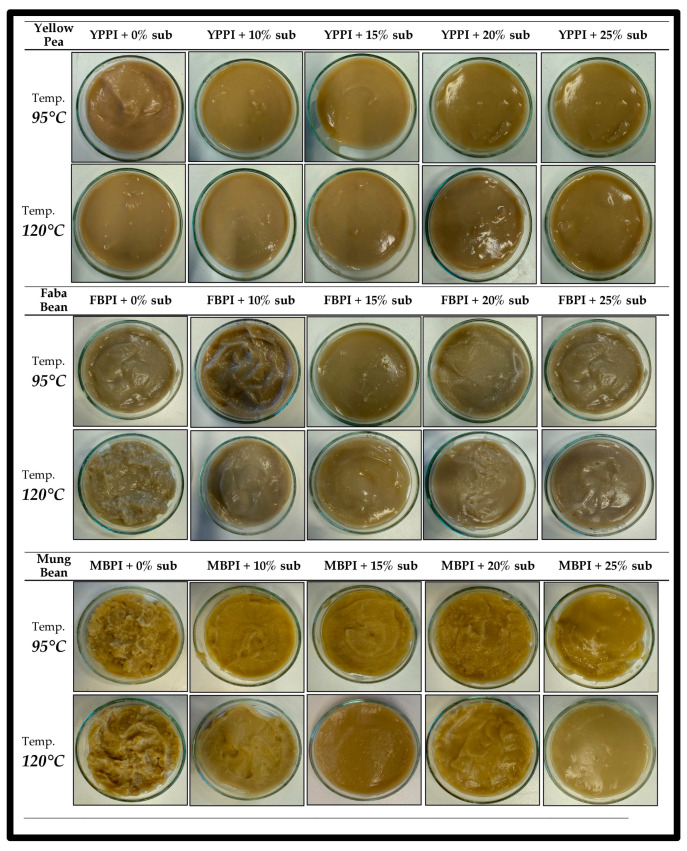
Qualitative photographs of post-RVA samples after heating and handling, illustrating visual differences in aggregate appearance across legume systems and starch substitution levels.

Despite these heating-related limitations, starch substitution produced a clear and consistent separation between the protein-only and 25% starch formulations in the yellow pea system, with significantly higher peak and final viscosities observed at increased starch levels (*p* < 0.05). This separation was more clearly resolved than in faba bean systems and more controlled than the starch-dominated response observed in mungbean systems. The behaviour likely reflects the mixed globulin composition of yellow pea proteins; although vicilin dominates, the presence of legumin contributes to a supportive protein framework that moderates starch swelling and reassociation under severe thermal conditions. These differences were also apparent from a qualitative visual observation of post-RVA aggregates (Figure 2), which illustrates the differences in sample appearance after processing and handling. With increasing starch substitution, aggregation across all legumes became more cohesive and continuous, consistent with previous reports on cooked, aggregated structures in protein-only legume systems following severe thermal or thermo-mechanical processing [3,19,35,36].

Faba bean systems exhibited the most stable and structurally reinforced pasting behaviour among the three legumes. At 95 °C, peak viscosity increased with starch substitution (2058 to 3018 cP; Figure 1c), while trough viscosity remained comparatively high (Table 2). A key advantage of the faba bean system was its ability to maintain structural integrity during heating and holding. This was evident from the sustained viscosity from peak through most of the holding phase, with a noticeable decrease occurring only toward the end of holding. This stability reflects the dominance of legumin (11S globulin), a slow-unfolding, disulfide-rich protein known to form heat-induced aggregates that reinforce granule swelling during gelatinization [37,38]. Earlier work has similarly shown that legumin-rich systems provide stronger paste stability than vicilin-dominant proteins due to their more cohesive unfolding and aggregation pathways [3,19,39].

A distinctive feature of the faba bean profiles was the emergence of a small shoulder before the main peak with starch addition, a behaviour absent in yellow pea and mungbean systems (Figure 1c,d). The presence of this shoulder suggests overlapping contributions from two or more components with different gelatinisation or swelling behaviours during the holding phase, rather than a single discrete gelatinisation event [40]. Similar shoulder or inflexion features in RVA pasting curves have been reported in complex starch systems containing components with different gelatinisation characteristics. Such behaviour likely reflects intrinsic differences in starch granule structure and thermal heterogeneity within the faba bean system. While the shoulder is primarily starch-driven, its expression occurs within a protein-rich matrix dominated by legumin, which may modulate water distribution and mechanical constraint during early swelling, thereby influencing the development of the main viscosity peak [41,42].

At 120 °C, faba bean maintained its superior structural resilience (Figure 1d). Peak viscosity remained high (3200 to 4040.5 cP), and breakdown was notably lower than in yellow pea and mungbean, confirming that legumin’s higher denaturation enthalpy and stronger aggregation behaviour allow it to stabilise granules under severe thermal stress. This performance is consistent with previous findings that legumin rich mixed with starch form heat-stable aggregates that reinforce starch-based pastes at temperatures relevant to extrusion and high-shear processing [3,6,19,43,44].

Final viscosity behaviour further highlighted faba bean’s strong structural contribution. At 95 °C, the final viscosity remained steady across substitution levels, reflecting controlled network formation during cooling. At 120 °C, the final viscosity increased sharply at higher substitution levels, suggesting that starch availability offsets granule deformation caused by high-temperature shear. This agrees with previous reports showing that legumin interacts with amylose to promote stronger retrograded networks during cooling, producing firmer gels than pea or mungbean systems [45,46,47].

Mungbean systems displayed a pasting pattern distinct from yellow pea and faba bean. At 95 °C, peak viscosity increased sharply with starch substitution (3886 to 8696 cP; Figure 1e, Table 3), reflecting the exceptionally high amylose content and strong water-binding capacity of mungbean starch [48]. Similar steep increases in viscosity have been widely reported for mungbean starch, which forms dense, swollen granules with limited permeability during gelatinization [49]. The combination of high amylose and compact granule morphology produces pastes that thicken rapidly but are comparatively brittle under shear.

Despite the strong initial viscosity development, mungbean pastes exhibited poor stability, indicated by high breakdown values and substantial trough reductions (Table 1). These findings suggest that mungbean protein contributes minimally to granule reinforcement during heating. Mungbean globulins unfold more gradually and possess weaker aggregation tendencies than legumin-rich faba bean proteins, resulting in limited capacity to stabilise swollen granules at peak swelling [49]. Previous work has similarly shown that mungbean proteins interact weakly with starch matrices and contribute less to viscosity maintenance during high-temperature swelling [50,51].

At 120 °C, the instability became more pronounced (Figure 1f). Although peak viscosity continued to rise with starch substitution (7204 to 8913 cP), breakdown remained high for all treatments, and trough viscosities dropped substantially. Severe thermal stress at 120 °C accelerates granule rupture and amylose leaching, particularly in amylose-rich starches such as mungbean [52,53,54]. The limited stabilisation from the protein fraction allows for a rapid granule deformation, explaining the sharp trough decline and reduced paste integrity. These observations agree with high-temperature studies indicating that mungbean starch loses structural cohesion more readily than pea or faba bean varieties during extreme heating [55].

Final viscosity trends also reflected the fragile structuring behaviour of mungbean systems. At 95 °C, the final viscosity decreased progressively at higher substitution levels, suggesting an inefficient reassociation of amylose and limited involvement of protein in forming a continuous network during cooling. At 120 °C, the final viscosity was even lower and more variable, consistent with extensive granule damage and weak retrogradation potential. The effect is mainly driven by starch granule rupture and loss of structural integrity during heating [55,56,57].

Overall, the RVA results revealed distinct, legume-specific pasting behaviours. Yellow pea systems were distinctive at 95 °C, where viscosity increased after breakdown and rose to a higher final viscosity during cooling, consistent with starch reassociation during cooling in starch-containing systems. Faba bean systems exhibited the most stable pasting profiles across temperatures, with a lower breakdown and more consistent final viscosity, reflecting the thermal robustness of the legumin-rich protein matrix. Mungbean systems, particularly with starch substitution, generated the highest peak viscosity at 95 °C, highlighting the strong swelling and amylose-driven thickening capacity of mmungbeanstarch. However, this structure was temperature-sensitive and became unstable at 120 °C, showing irregular profiles and limited viscosity recovery. This analysis does not reproduce the complex shear fields and residence times characteristic of industrial processing; it provides a controlled high-temperature and moderate-shear environment for comparative assessment of formulation-dependent viscosity development and structural development during heating and cooling. High-temperature RVA measurements therefore offer practical and mechanistic insight into ingredient performance under industrially relevant thermal conditions, supporting formulation screening and interpretation of processing-related structural trends [24,58,59].

### 2.2. Gel Strength

**Figure 3:**
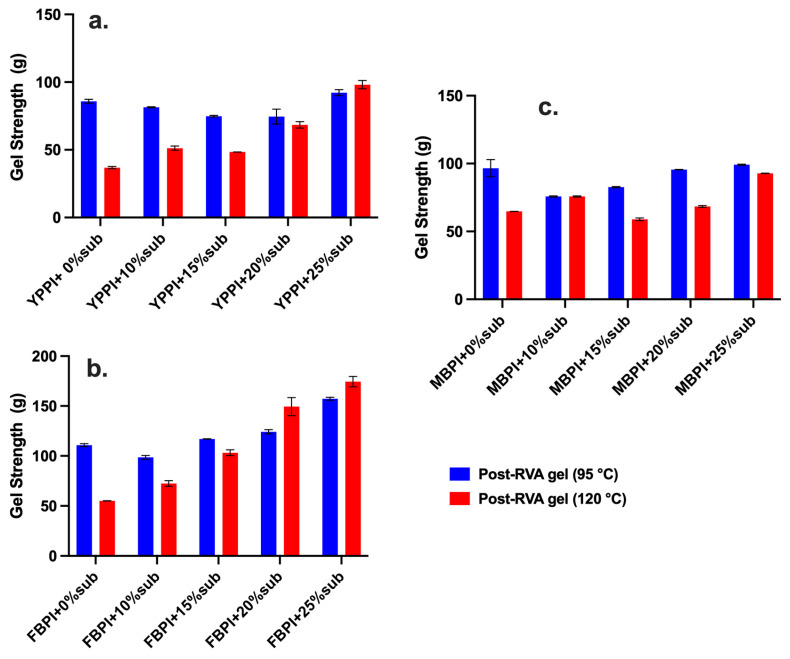
Gel strength of legume protein–starch systems across 0–25% (*w*/*w*) substitution following RVA processing: (**a**) yellow pea; (**b**) faba bean; and (**c**) mungbean, measured at 95 °C and 120 °C to assess differences in network formation and thermal reinforcement.

Gel strength is one of the most informative indicators of the functional performance of protein–starch systems, as it reflects the ability of the matrix to establish a cohesive three-dimensional network during cooling [60]. Gel strength measurements were performed on samples after RVA heating and cooling and therefore reflect the mechanical integrity of the final, consolidated protein–starch network rather than gel formation during heating. When interpreted alongside RVA pasting behaviour and temperature-ramp rheology, gel strength provides an integrated measure of how thermal processing and starch substitution translate into final network robustness across different legume systems. As a post-processing textural parameter, gel strength is widely used to assess the robustness of composite food networks and the cumulative effects of protein aggregation and starch retrogradation following thermal treatment [8,61]. Gel-strength measurements assessed via texture analysis (see Figure 3 and Table 1, Table 2 and Table 3) revealed distinctly different structuring capacities among the three legume systems. The gel-strength behaviour of yellow pea closely mirrored the trends observed in the RVA profiles (Figure 3a). At 95 °C, gels remained weak across 0–20% substitution, consistent with the limited viscosity stability and pronounced breakdown seen during heating (Figure 1a). These outcomes reflect the early unfolding and low interaction capacity of vicilin, which provides little structural reinforcement during gelatinisation and leaves the system poorly structured for cooling-driven network consolidation [62]. As a result, gel strength varied only slightly across the lower substitution levels, indicating that modest increases in starch content were insufficient to offset the weak protein matrix.

A more noticeable improvement appeared only at 25%, where gel strength increased in parallel with the rise in final viscosity. This behaviour suggests that once amylose availability reaches a sufficient threshold, network reinforcement becomes predominantly starch-driven. This is also evident in the RVA curves, where the 0–20% substitutions follow highly similar collapse-and-recovery patterns, whereas the 25% sample shows a distinct enhancement in the final viscosity at both temperatures (Figure 1a,b). A similar report shows that pea-based systems may exhibit increased viscosity through starch recrystallisation without forming strong, protein-supported gels, a behaviour also evident here [18]. Thus, yellow pea gels remained mechanically fragile despite the higher firmness at 25%, underscoring the minor contribution of vicilin to junction-zone formation during cooling.

At 120 °C, the weak structuring tendency of yellow pea became even more apparent. Across 0–20%, gel strength increased only gradually, reflecting the highly disrupted paste structures produced under severe heating (Figure 3b). The lack of protein-mediated stabilisation at this temperature limited the system’s ability to recover during cooling, resulting in gels that remained soft despite differences in starch content [10]. As at 95 °C, a firmer gel emerged only at 25%, where extensive amylose release during high-temperature treatment likely enhanced retrogradation and strengthened the final network.

Faba bean exhibited the strongest final gel strength among the three legumes. At both 95 °C and 120 °C, gel strength increased consistently with starch substitution, reaching 174.39 g at the highest substitution level under 120 °C (Figure 3b). The gel strength of the faba bean samples increased with starch substitution and remained consistently high. This pattern reflects the capacity of legumin to participate actively in network formation during cooling, which is similar to the strengthened mixed gels reported by Huang et al. [9]. The strong gelation response also aligns with the pasting behaviour, where faba bean demonstrated the most stable viscosity profiles, low breakdown, and clear structural reinforcement during heating (Figure 1c,d). These behaviours arise from its legumin rich protein composition. Legumin unfolds more slowly, possesses abundant sulfhydryl groups, and forms dense heat-induced aggregates that stabilise swollen granules and support network formation during cooling [47]. These aggregates provide a structural framework that enhances amylose and amylopectin associations, resulting in cohesive and elastic gels.

Mungbean displayed intermediate but distinctive behaviour, consistent with its high-amylose starch composition. At 95 °C, gel strength decreased initially (10–15%) before rising sharply at 20–25%, indicating that the early dilution of the protein network reduced structural cohesion until starch became sufficiently dominant to drive network strengthening through amylose retrogradation. A similar trend was observed at 120 °C, where gel strength remained modest at low substitution but increased substantially at 25%. High-temperature amylose leaching is known to promote firm gel networks upon cooling, provided that sufficient amylose is available and granule remnants remain capable of contributing to junction zone formation [26]. However, the overall gel strength remained lower than faba bean, reflecting the relatively weaker aggregation capacity of mungbean proteins and the limited protein–polysaccharide reinforcement observed in the pasting stage (Figure 1e,f).

Overall, these results show that gel structure development is legume-specific, with yellow pea relying mainly on starch, mungbean strengthened by amylose at higher substitution, and faba bean forming the most robust protein–starch networks.

### 2.3. Temperature Ramp

Temperature-ramp rheology was performed on all systems at a total solids concentration of 10% (*w*/*w*), which is sufficient for proteins to form a continuous network prior to heating. For starch-substituted formulations, only the 25% (*w*/*w*) level was included in the rheological analysis. Rheological measurements were therefore conducted at two extreme starch substitution levels (0% and 25%) to enable a direct comparison between protein-only systems and formulations containing a substantial starch fraction. Temperature-dependent rheology was used to support and mechanistically interpret trends identified by RVA, which was performed across all sample combinations, rather than to assess incremental compositional effects. Previous studies on high-temperature protein–starch systems have shown that incremental increases in starch content primarily modulate pasting behaviour and bulk textural responses without introducing additional structural transitions once protein-dominated systems are established [19]. On this basis, an endpoint-based temperature-ramp rheological approach was adopted to examine the network development during heating.

In this context, the gradual changes observed at intermediate starch levels in the RVA and gel strength measurements support the use of temperature-ramp rheology as an endpoint mechanistic comparison, while recognising that finer compositional resolution would be required to fully describe progressive structural transitions.

Accordingly, the observed rheological behaviour reflects the reinforcement of an already elastic network rather than a sol–gel transition, as G′ remained greater than G″ throughout the temperature sweep. The pre-existing elastic character observed across all systems may be attributed, at least in part, to the industrial processing history of the commercial protein isolates, which can result in the partial unfolding and aggregation before thermal analysis, as widely reported for commercial plant protein powders [63,64,65,66].

**Figure 4:**
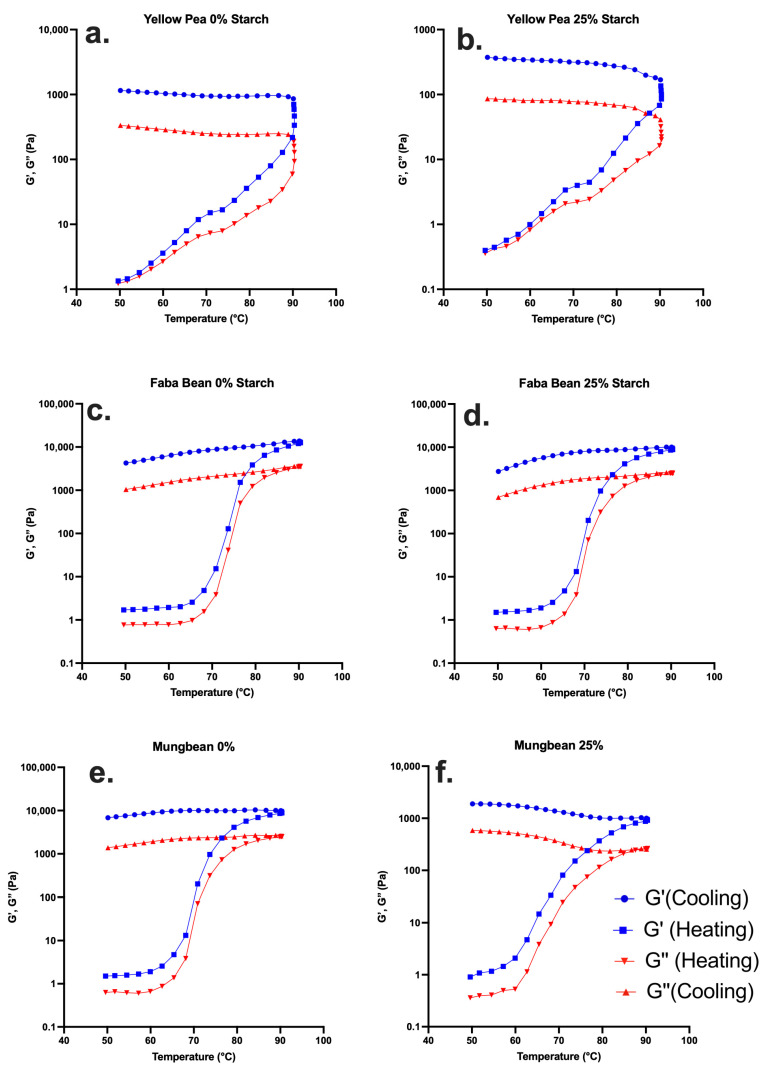
Temperature-ramp rheology of (**a**,**b**) yellow pea, (**c**,**d**) faba bean, and (**e**,**f**) mungbean protein–starch systems at 0% (*w*/*w*) and 25% (*w*/*w*) starch. G′ represents the storage modulus, and G″ represents the loss modulus.

Yellow pea exhibited the lowest elastic moduli of the three legumes. In the 0% (*w*/*w*) starch system (Figure 4a), G′ increased only modestly during heating, reflecting the gradual unfolding and aggregation of vicilin-rich proteins. Vicilin contains few sulfhydryl groups and fewer reactive hydrophobic domains, which limits its ability to form strong intermolecular associations. Consequently, the plateau G′ observed during the high-temperature holding phase remained low and corresponded to the weak gel strengths reported for yellow pea in Table 1. Substituting 25% (*w*/*w*) starch (Figure 4b) accelerated the modulus development because swollen starch granules contributed to the early increases in network rigidity. However, the final G′ remained comparatively low, indicating that starch addition could not compensate for the weak protein matrix. This response reflects the limited protein–starch structural synergy, consistent with earlier studies showing that vicilin interacts only minimally with granule surfaces and provides minimal reinforcement to composite networks [28].

Faba bean displayed the strongest elastic behaviour among the three legumes. In the 0% (*w*/*w*) starch system (Figure 4c), G′ increased sharply during heating and reached plateau values an order of magnitude higher than those of yellow pea. This pattern is characteristic of legumin-dominant proteins, which unfold gradually and form densely aggregated networks through disulfide interchange and hydrophobic association [3]. Similar rapid G′ development in concentrated legume protein systems has been reported previously, reflecting the high aggregation capacity and structural rigidity of legumin-rich matrices [6,19]. In the 25% (*w*/*w*) starch system (Figure 4d), the modulus development intensified further. Swollen starch granules acted as reinforcing mechanical fillers within the protein network, leading to a substantial increase in the final plateau modulus during both heating and cooling [14]. This strong protein and starch coupling aligns with the exceptionally high gel strengths measured for faba bean and with the stable, low-breakdown RVA behaviour.

Mungbean showed intermediate viscoelastic behaviour, with G′ values consistently higher than yellow pea but still below those of faba bean. In the 0% (*w*/*w*) starch system (Figure 4e), G′ increased steadily during heating, reflecting the combined effects of protein unfolding and amylose-rich starch swelling. Although this early modulus development was notable, the plateau values remained lower than those of faba bean, indicating a less cohesive protein network. With 25% (*w*/*w*) starch (Figure 4f), G’ increased non-linearly during heating, with a gradual rise at lower temperatures followed by a steeper increase at higher temperatures. Despite this strengthening during heating, G′ retention during cooling remained modest, consistent with the lower gel strengths observed in Table 1. The limited recovery is supported by earlier studies describing mungbean starch and protein matrices as prone to irreversible breakdown under shear, thixotropic flow, and weaker reorganisation during cooling [3,45,67,68].

Based on the temperature-ramp results, faba bean showed the strongest thermal strengthening, with G′ increasing sharply during heating and recovering well during cooling, indicating the formation of a dense and stable elastic network. Mungbean exhibited moderate strengthening with only partial recovery, while yellow pea showed the weakest and least cohesive viscoelastic development, confirming its limited ability to form heat-stable structures. The observed rheological behaviour reflects reinforcement of an already elastic network rather than a sol–gel transition, as G′ remained greater than G″ throughout the temperature sweep. The pre-existing elastic character observed across all systems may be attributed, at least in part, to the industrial processing history of the commercial protein isolates, which can result in partial unfolding and aggregation before thermal analysis, as widely reported for commercial plant protein powders [63,64,65].

### 2.4. Differential Scanning Calorimetry (DSC)

**Figure 5:**
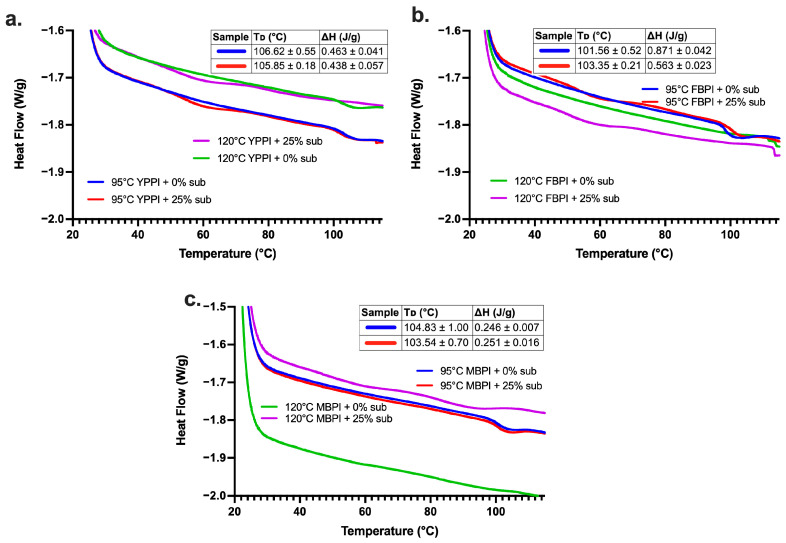
DSC thermograms of freeze-dried RVA gels for (**a**) yellow pea, (**b**) faba bean, and (**c**) mungbean protein–starch systems at 0% (*w*/*w*) and 25% (*w*/*w*) substitution.

While oscillatory rheology describes the mechanical consequence of these transformations, it does not reveal the extent of residual molecular order retained after heating. To clarify the extent to which thermally responsive protein or starch domains remain after RVA processing, DSC was conducted on freeze-dried RVA gels following the post-pasting approach of [19]. It is important to note that DSC measurements reflect residual meltable domains remaining after thermal processing rather than native ordered structures present prior to heating. This resulting endothermic transition therefore provides information on the extent to which reorganised or aggregated domains remain thermally responsive. At 95 °C (Figure 5a), yellow pea exhibited small and closely spaced enthalpy values, decreasing only marginally from 0.463 J/g (0% (*w*/*w*)) to 0.438 J/g (25% (*w*/*w*)). This limited enthalpy change indicates that very little ordered structure was rebuilt after RVA heating, even when starch substitution increased. Yellow pea is dominated by vicilin, a trimeric globulin with low sulfhydryl content and poor aggregation behaviour, which rapidly unfolds during heating and exhibits minimal capacity to form stable ordered domains upon cooling [66]. Accordingly, the observed transitions are interpreted as weak residual ordering dominated by starch-associated domains rather than protein-derived meltable structures.

This behaviour agrees with reports on other vicilin-rich systems, such as lentil and chickpea, where DSC enthalpy remained consistently low (<0.6 J/g) after shear-intensive thermal processing, and starch addition produced only minor modulation [28]. Similar findings have been reported for pea protein–starch mixtures, where amylose retrogradation remained weak due to extensive granule disruption and limited protein reinforcement [69]. At 120 °C, the DSC curves flattened further and lacked distinct peaks, reflecting the near-complete loss of residual meltable domains following severe RVA heating, consistent with previous high-temperature pea studies showing that vicilin-dominant systems lose all thermally responsive structure under extreme thermo-mechanical conditions [3,28].

Among the three legumes, faba bean exhibited the highest enthalpy change at 95 °C (Figure 5b), with ΔH decreasing from 0.871 J g^−1^ at 0% substitution to 0.563 J g^−1^ at 25%. The relatively high enthalpy at low substitution levels is consistent with the thermal behaviour of legumin-rich systems, as legumin (11S globulin) is a thermally stable, hexameric storage protein whose denaturation requires substantial energy [5]. In this context, ΔH is interpreted as reflecting the residual meltable protein-associated domains rather than an intact native protein structure. The progressive reduction in ΔH with increasing starch substitution suggests a modification of protein aggregation behaviour within the mixed matrix, where starch can compete for water, act as a dispersed filler, and promote partial phase separation, thereby limiting the formation or stability of meltable, ordered protein aggregates [14]. At 120 °C, DSC thermograms became largely featureless across all formulations, reflecting extensive denaturation and irreversible aggregation of legume globulins into non-meltable structures, which eliminates detectable thermal transitions at elevated temperature.

Mungbean systems exhibited only small endothermic transitions across all treatments, with low ΔH values and onset temperatures above 100 °C (Figure 5c), indicating limited residual ordered structure following RVA processing. While higher denaturation enthalpies have been reported for raw or minimally processed mungbean protein isolates, extensive thermal and mechanical processing is known to substantially reduce or eliminate detectable DSC transitions in legume proteins [3,48]. This response is consistent with the protein composition of mungbean, which is dominated by vicilin-type 8S globulins that generally exhibit lower thermal stability and weaker aggregation capacity than legumin-rich systems [7,70]. In parallel, mungbean starch undergoes rapid crystalline disruption at moderate temperatures and retains limited residual gelatinisation enthalpy after processing, further contributing to the low DSC signal observed [48,71]. Following the 120 °C RVA treatment, thermograms became flatter with reduced heat-flow intensity, reflecting extensive and irreversible denaturation of both protein and starch domains under severe thermal conditions. The absence of a discernible ΔH difference between 0% and 25% starch formulations suggests that additional starch did not contribute measurably to the formation of reorganised, meltable domains during cooling, reinforcing that mungbean matrices exhibit limited structural recovery after high-temperature processing.

Across all legumes, DSC confirmed that higher RVA processing severity consistently reduced residual molecular order, with 120 °C treatments producing flatter thermograms and markedly lower enthalpy than those processed at 95 °C. The magnitude of this loss differed by protein type: legumin-rich faba bean retained the greatest ordered structure, yellow pea showed minimal recovery likely due to weak vicilin aggregation, and mungbean exhibited the lowest extent of residual meltable domains overall, reinforcing the rheological and RVA evidence that these systems diverge strongly in their ability to rebuild structures after high-temperature shear.

From an application perspective, the strong thermal stability and gelation capacity of faba bean make it well-suited for structured products such as plant-based meat analogues, retorted foods, and extrusion-based applications. Yellow pea, which provides moderate viscosity with temperature-dependent stability enhanced by starch, is better suited for soups, sauces, beverages, and dairy-alternative systems. Mungbean, characterised by rapid viscosity development but limited structural recovery, is most appropriate for starch-driven products such as noodles and vermicelli or applications requiring rapid thickening without strong gel formation. These application-oriented interpretations, derived from model processing conditions, highlight the need for future work to tailor performance through targeted processing and formulation strategies, particularly starch–protein interactions and hybrid legume systems.

## 3. Conclusions

This study demonstrates that the thermal and structural behaviours of legume protein–starch systems differ markedly among yellow pea, faba bean, and mungbean, with these differences consistently reflected across RVA pasting profiles, temperature-ramp rheology, gel strength measurements, and DSC transition.

Yellow pea systems exhibited a temperature-sensitive pasting response consistent with behaviour commonly reported for vicilin-rich legume protein systems in the literature. At 95 °C, controlled viscosity development and effective recovery during cooling were observed, while at 120 °C protein-only systems showed a pronounced breakdown that was partially mitigated by starch addition. Rheological and gel strength measurements indicated moderate elastic network reinforcement, and DSC showed limited preservation of residual ordered domains following heating.

In contrast, faba bean systems exhibited the most cohesive and thermally robust response across all conditions. This behaviour aligns with the literature-reported patterns for legumin-rich systems, characterised by sustained viscosity during holding, lower breakdown, dominant elastic behaviour, and higher gel strength. RVA and DSC results further indicated an effective stabilisation of swollen granules and the presence of thermally resilient residual domains, identifying faba bean as the most structurally stable system under both moderate and high-temperature processing.

Mungbean systems generated high peak viscosities driven by starch but showed limited protein-mediated stabilisation, resulting in pronounced breakdown and poor viscosity retention, particularly at 120 °C. Rheological measurements indicated weak elastic reinforcement, and DSC revealed minimal residual structural order after severe heating, indicating a predominantly starch-dominated and thermally unstable structural response.

These results clarify how protein type and starch substitution influence high-temperature gelation, guiding the development of heat-stable plant-based gels.

## 4. Materials and Methods

### 4.1. Material

Yellow pea protein isolate (YPEAPR01), mungbean protein isolate (MUBNPR01), and faba bean protein isolate (FABRPRO03) were supplied by Australian Plant Proteins (Horsham, VIC, Australia). All protein isolates were produced via proprietary wet fractionation processes. Based on the manufacturer’s typical nutritional information, the yellow pea, mungbean, and faba bean protein isolates contained 83.0, 84.8, and 87.6 g protein per 100 g, respectively, on an as-is basis. All isolates were non-GMO, suitable for vegan applications, and sourced and manufactured in Australia. According to supplier specifications, the isolates exhibited good aqueous solubility at neutral pH (approximately 75–87% at pH 7), reflecting their suitability for hydrated protein systems and thermal processing.

The corresponding dry-fractionated starches from yellow pea and faba bean were supplied as Essantis Pea Starch 60 and Essantis Faba Bean Starch 60 (Essantis Pty Ltd., Newtown, VIC, Australia). According to the manufacturer, these starch fractions contained approximately 60% (*w*/*w*) starch and were obtained via dry fractionation, preserving the native granule structure and avoiding chemical modification. For the mungbean system, the starch fraction was provided by the Australian Export Grains Innovation Centre (AEGIC) (Sydney, NSW, Australia).

Analytical-grade sodium chloride (NaCl), sodium phosphate monobasic dihydrate (NaH_2_PO_4_·2H_2_O), and sodium phosphate dibasic dihydrate (Na_2_HPO_4_·2H_2_O) were purchased from Sigma-Aldrich (Darmstadt, Germany). Milli-Q™ water (Millipore SAS, Molsheim, France) was used for all sample preparation and buffer solutions.

### 4.2. Methods

#### 4.2.1. Sample Preparation

Protein–starch mixtures were prepared following the method described in [19] with modification. An initial trial was conducted to determine a solids concentration suitable for high-temperature RVA analysis. While formulations in previous study [19] at 20% (*w*/*w*) have been used for laboratory-extracted proteins, applying this concentration to the commercial protein isolates used here produced excessively viscous, non-flowable dispersions that interfered with mixing and paddle rotation. Reducing the solids content to 17% (*w*/*w*) yielded fully hydrated, homogeneous dispersions that remained flowable throughout heating while still providing sufficient viscosity development. All formulations were prepared at a constant total solids content of 17% (*w*/*w*). Protein was partially replaced by starch on a weight-for-weight basis whereby starch replaced an equivalent mass of protein while maintaining a constant total solid with substitution levels ranging from 0 to 25% (*w*/*w*) (%*w*/*w*), as summarised in Table 4.

Following formulation, all dispersions were hydrated overnight at 4 °C to allow for a complete water uptake by both protein and starch fractions. On the following day, samples were mixed on a tube roller for 1 h to ensure uniform dispersion and to minimise local concentration gradients. Immediately prior to RVA analysis, each sample was further homogenised using magnetic stirring for 10 min to break up any remaining agglomerates and ensure consistent initial viscosity at the start of heating.

This concentration represented the highest level at which yellow pea, faba bean, and mungbean systems all exhibited stable pre-heating viscosity, reproducible pasting curves, and no mechanical interference during RVA operation, ensuring the meaningful comparison of intrinsic protein–starch interactions.

#### 4.2.2. Rapid Visco Analyser (RVA)

The pasting behaviour of the prepared protein–starch mixtures (Section 4.2.1) was characterised using a high-temperature Rapid Visco Analyzer (RVA) 4800 by Perten Instruments Australia Pty Limited, Sydney, NSW, Australia. This instrument enables the viscosity analysis at temperatures exceeding the boiling point of water under controlled shear and thermal conditions, allowing for an evaluation of ingredient functionality under processing-relevant temperatures above 100 °C. At temperatures above 100 °C, the RVA 4800 operates as a sealed and self-pressurised system, in which the canister is secured by an O-ring-sealed lid and pressurised during heating. This configuration prevents sample boiling and vapour formation, maintaining a stable liquid phase during high-temperature measurements and enabling reliable viscosity recording up to 140 °C. Heating temperature and paddle rotation are computer-controlled, ensuring reproducible thermal and shear histories across measurements [59,72]. Each sample was loaded into the RVA canister and analysed under two heating programmes representing moderate (95 °C) and high-temperature (120 °C) processing. The 95 °C pasting test followed the temperature–time profile of the AACC Standard Method 76–21.02, consisting of an initial hold at 50 °C for 1 min, heating to 95 °C over 3 min 42 s, holding at 95 °C for 2 min 30 s, cooling to 50 °C over 3 min 48 s, and a final 3 min hold at 50 °C. The high-temperature pasting test (120 °C) followed the protocol described by Devkota et al. [19] using an initial hold at 50 °C for 1 min, heating to 120 °C over 5 min 50 s, holding at 120 °C for 2 min 30 s, cooling to 50 °C over 5 min 50 s, and a final 3 min hold at 50 °C. The mixing speed for all tests was maintained at 160 rpm after an initial 10 s dispersion at 960 rpm.

#### 4.2.3. Gel Strength Analysis

The mechanical strength of the protein and protein–starch gels was determined using a Texture Analyzer (TA.XTplusC, Stable Micro Systems, UK) fitted with a 10 mm cylindrical probe (P/10). Gels obtained from the RVA pastes prepared at 95 °C and 120 °C were stored at 4 °C for 24 h. This resting period was selected based on previous studies on legume protein and protein–starch gels, in which refrigerated storage is used to allow completion of cooling-induced network consolidation and starch retrogradation before texture analysis [19]. All samples were equilibrated to room temperature prior to testing to ensure consistent measurement conditions. Gel strength was determined as the peak force obtained during the first compression to a deformation depth of 10 mm.

#### 4.2.4. Thermal Transition (DSC)

Thermal analyses were performed using a DSC 2500 (TA Instruments, New Castle, DE, USA). All samples originated from RVA-heated gels processed at 95 °C and 120 °C, which were subsequently freeze-dried (FreeZone, Labconco, Kansas City, MO, USA), ground into powder, and sealed in aluminium pans containing 10 μL of Milli-Q water. This post-pasting DSC approach was selected following the methodology reported by Devkota et al. [19], in which DSC is applied after thermal–shear treatment to probe residual meltable domains remaining after processing rather than native protein or starch structures. Approximately 5 mg of the sample was used for each run. The pans were equilibrated at 4 °C overnight to allow uniform moisture redistribution prior to scanning. DSC measurements were carried out from 20 °C to 115 °C at a heating rate of 10 °C min^−1^ under a nitrogen purge, with an empty sealed pan used as the reference. Thermograms were processed using TA Trios software (version 5) to determine the onset temperature (*T*_o_), peak temperature (*T*_p_), conclusion temperature (*T*_c_) and Enthalpy (ΔH).

#### 4.2.5. Temperature Ramp

Samples were prepared at 10% (*w*/*w*) *w*/*w* solids by dispersing 1.1 g of material into 10 mL phosphate-buffered saline (PBS). Each legume system (yellow pea, faba bean, and mungbean) was formulated at two substitution levels. For the 0% (*w*/*w*) starch samples, all solids consisted of protein isolate (1.1 g). For the 25% (*w*/*w*) starch substitution, 0.83 g of protein isolate and 0.27 g of the corresponding dry-fractionated starch fraction were combined to obtain 1.1 g of total solids. All dispersions were hydrated overnight at 4 °C to ensure complete water uptake, then mixed on a tube roller for 1 h prior to analysis.

Rheological measurements were performed using a strain-controlled ARES G2 rheometer (TA Instruments, New Castle, DE, USA) equipped with a 50 mm parallel plate geometry. Approximately 2 mL of sample was loaded onto the lower plate maintained at 25 °C, and the upper plate was lowered to a 1 mm gap, with excess material trimmed to minimise edge effects. Temperature sweep tests were conducted under small-amplitude oscillatory shear within the linear viscoelastic region, using a constant strain of 1% and an angular frequency of 1 rad s^−1^, where the storage modulus (G′) represents the elastic response and the loss modulus (G″) the viscous response of the gels [29]. Samples were heated from 50 °C to 95 °C, held at 90 °C for 30 min, and cooled to 50 °C at a constant heating and cooling rate of 2 °C min^−1^, following established protocols for pulse protein systems [3].

#### 4.2.6. Data Analysis

All experiments were conducted in duplicate and results are reported as mean ± standard deviation. Statistical analyses were performed using IBM SPSS Statistics version 30.0. One-way ANOVA was applied to assess the effect of starch substitution level within each legume type and temperature condition. Tukey’s B multiple-comparison test was used to identify statistically significant differences at *p* < 0.05. Graphs and visualisations were produced using GraphPad Prism version 10.

## Figures and Tables

**Table 1:** Summary of functional properties (viscosity and gel strength) of yellow pea protein–starch system.

(a) RVA Parameters and Gel Strength at 95 °C Heating					
Parameter	YPPI + 0% Sub	YPPI + 10% Sub	YPPI + 15% Sub	YPPI + 20% Sub	YPPI + 25% Sub
Peak (cP)	1814.0 ± 1.0 ^a^	1949.5 ± 17.5 ^b^	2053.0 ± 35.0 ^c^	2277.0 ± 5.0 ^d^	2461.5 ± 33.5 ^e^
Trough (cP)	1581.5 ± 9.5 ^a^	1804.5 ± 24.5 ^b^	1909.0 ± 30.0 ^c^	2141.0 ± 1.0 ^d^	2331.5 ± 30.5 ^e^
Breakdown (cP)	232.5 ± 8.5 ^a^	145.0 ± 42.0 ^a^	144.0 ± 5.0 ^a^	136.0 ± 4.0 ^a^	130.0 ± 3.0 ^a^
Final Viscosity (cP)	2472.0 ± 83.0 ^a^	2718.0 ± 88.0 ^ab^	2742.5 ± 26.5 ^b^	3023.5 ± 31.5 ^c^	3292.5 ± 91.5 ^d^
Setback (cP)	890.5 ± 73.5 ^a^	913.5 ± 112.5 ^a^	833.5 ± 56.5 ^a^	882.5 ± 30.5 ^a^	961.0 ± 61.0 ^a^
Gel Strength (g)	85.8 ± 1.5 ^b^	81.4 ± 0.4 ^ab^	74.8 ± 0.6 ^a^	74.5 ± 5.5 ^a^	92.2 ± 2.2 ^c^
**(b) RVA Parameters and Gel Strength at 120 °C Heating**					
Peak (cP)	1990.0 ± 60.0 ^a^	2320.5 ± 25.5 ^a^	2324.5 ± 41.5 ^a^	2375.5 ± 418.5 ^a^	2781.5 ± 19.5 ^a^
Trough (cP)	723.5 ± 22.5 ^a^	907.0 ± 4.0 ^a^	862.5 ± 13.5 ^a^	952.5 ± 200.5 ^a^	1186.5 ± 9.5 ^a^
Breakdown (cP)	1266.5 ± 37.5 ^a^	1413.5 ± 21.5 ^a^	1462.0 ± 28.0 ^a^	1423.0 ± 218.0 ^a^	1595.0 ± 10.0 ^a^
Final Viscosity (cP)	1639.5 ± 74.5 ^a^	2060.0 ± 7.0 ^a^	1891.0 ± 38.0 ^a^	1931.5 ± 401.5 ^a^	2450.0 ± 18.0 ^a^
Setback (cP)	916.0 ± 52.0 ^a^	1153.0 ± 3.0 ^a^	1028.5 ± 24.5 ^a^	979.0 ± 201.0 ^a^	1263.5 ± 8.5 ^a^
Gel strength (g)	36.8 ± 0.8 ^a^	51.2 ± 1.5 ^b^	48.4 ± 0.1 ^b^	68.5 ± 2.4 ^c^	98.1 ± 3.1 ^d^

Values are expressed as mean ± standard deviation (*n* = 2). Within each row and temperature, means followed by different superscript letters indicate significant differences (*p* < 0.05) according to Tukey’s B multiple-comparison test.

**Table 2:** Summary of functional properties (viscosity and gel strength) of faba bean protein–starch system.

(a) RVA Parameters and Gel Strength at 95 °C Heating					
Parameter	FBPI + 0% Sub	FBPI + 10% Sub	FBPI + 15% Sub	FBPI + 20% Sub	FBPI + 25% Sub
Peak (cP)	2058.0 ± 33.0 ^a^	2424.0 ± 42.0 ^b^	2819.0 ± 35.0 ^c^	2923.5 ± 41.5 ^c^	3018.0 ± 38.0 ^d^
Trough (cP)	1314.5 ± 22.5 ^a^	1752.5 ± 24.5 ^b^	2120.5 ± 30.0 ^c^	2211.0 ± 31.0 ^c^	2228.0 ± 33.0 ^c^
Breakdown (cP)	743.5 ± 37.5 ^a^	671.5 ± 21.5 ^a^	698.5 ± 28.0 ^a^	712.5 ± 21.8 ^a^	790.0 ± 10.0 ^a^
Final Viscosity (cP)	2531.0 ± 74.5 ^a^	2549.5 ± 7.0 ^a^	2570.0 ± 38.0 ^a^	2672.5 ± 41.5 ^a^	2642.0 ± 18.0 ^a^
Setback (cP)	1216.5 ± 52.0 ^c^	797.0 ± 3.0 ^b^	449.5 ± 24.5 ^a^	461.5 ± 30.5 ^a^	414.0 ± 8.5 ^a^
Gel Strength (g)	110.8 ± 1.5 ^b^	98.5 ± 0.4 ^a^	116.8 ± 0.6 ^b^	124.1 ± 5.5 ^c^	157.2 ± 2.2 ^d^
**(b) RVA Parameters and Gel Strength at 120 °C Heating**					
Peak (cP)	3200.5 ± 45.0 ^a^	3606.0 ± 38.0 ^ab^	3634.5 ± 41.5 ^ab^	4040.5 ± 52.0 ^b^	3772.0 ± 40.0 ^ab^
Trough (cP)	3200.5 ± 45.0 ^a^	3606.0 ± 38.0 ^ab^	3634.5 ± 41.5 ^ab^	4040.5 ± 52.0 ^b^	3772.0 ± 40.0 ^ab^
Breakdown (cP)	1995.5 ± 37.5 ^a^	2566.0 ± 21.5 ^b^	2709.5 ± 28.0 ^b^	3003.0 ± 31.0 ^c^	2986.0 ± 30.5 ^c^
Final Viscosity (cP)	3175.0 ± 52.0 ^c^	2574.5 ± 30.5 ^b^	1916.0 ± 24.5 ^a^	2117.0 ± 21.5 ^ab^	1815.0 ± 18.5 ^a^
Setback (cP)	1970.0 ± 61.0 ^c^	1534.5 ± 30.5 ^b^	991.0 ± 24.5 ^a^	1079.5 ± 30.5 ^a^	1029.0 ± 21.5 ^a^
Gel strength (g)	55.1 ± 1.0 ^a^	72.4 ± 1.5 ^b^	103.2 ± 2.4 ^c^	149.3 ± 3.1 ^d^	174.4 ± 3.5 ^e^

Values are expressed as mean ± standard deviation (*n* = 2). Within each row and temperature, means followed by different superscript letters indicate significant differences (*p* < 0.05) according to Tukey’s B multiple-comparison test.

**Table 3:** Summary of functional properties (viscosity and gel strength) of mungbean protein–starch system.

(a) RVA Parameters and Gel Strength at 95 °C Heating					
Parameter	MBPI + 0% Sub	MBPI + 10% Sub	MBPI + 15% Sub	MBPI + 20% Sub	MBPI + 25% Sub
Peak (cP)	3885.5 ± 108.0 ^a^	6707.5 ± 58.0 ^a^	6963.0 ± 325.0 ^b^	7480.5 ± 297.0 ^b^	8695.5 ± 200.0 ^c^
Trough (cP)	2655.5 ± 4.0 ^a^	4051.0 ± 156.0 ^ab^	3659.0 ± 141.0 ^b^	3719.5 ± 213.5 ^b^	4319.0 ± 100.0 ^b^
Breakdown (cP)	1230.0 ± 104.0 ^a^	2656.5 ± 97.0 ^ab^	3304.0 ± 184.0 ^b^	3761.0 ± 84.0 ^b^	4376.5 ± 100.0 ^c^
Final Viscosity (cP)	2721.5 ± 26.0 ^a^	5735.5 ± 115.0 ^a^	4994.5 ± 147.0 ^a^	5002.0 ± 75.0 ^b^	5611.5 ± 1.5 ^b^
Setback (cP)	66.0 ± 31.0 ^a^	1684.5 ± 40.0 ^a^	1335.5 ± 65.0 ^a^	1282.5 ± 138.5 ^a^	1292.5 ± 101.5 ^a^
Gel Strength (g)	96.6 ± 6.3 ^cd^	75.8 ± 0.4 ^a^	82.7 ± 0.4 ^ab^	95.6 ± 0.1 ^bc^	99.1 ± 0.4 ^d^
**(b) RVA Parameters and Gel Strength at 120 °C Heating**					
Peak (cP)	7204.0 ± 45.0 ^ab^	7623.0 ± 464.0 ^a^	7307.5 ± 31.0 ^b^	8010.5 ± 40.0 ^c^	8913.0 ± 291.0 ^ab^
Trough (cP)	492.5 ± 47.0 ^a^	581.5 ± 136.5 ^a^	653.0 ± 14.0 ^b^	901.0 ± 2.0 ^c^	1115.5 ± 256.0 ^a^
Breakdown (cP)	6711.5 ± 2.0 ^ab^	7041.5 ± 327.0 ^a^	6654.5 ± 17.0 ^b^	7109.5 ± 38.0 ^a^	7797.5 ± 34.0 ^ab^
Final Viscosity (cP)	4006.0 ± 84.0 ^a^	4648.5 ± 558.5 ^a^	5345.0 ± 295.0 ^a^	4133.5 ± 10.0 ^a^	3488.0 ± 605.0 ^a^
Setback (cP)	3513.5 ± 131.5 ^ab^	4067.0 ± 422.0 ^a^	4692.0 ± 309.0 ^a^	3232.5 ± 12.0 ^a^	2372.5 ± 348.0 ^ab^
Gel strength (g)	75.8 ± 0.1 ^d^	58.9 ± 0.4 ^a^	68.4 ± 1.0 ^c^	92.8 ± 0.7 ^e^	75.8 ± 0.1 ^d^

Values are expressed as mean ± standard deviation (*n* = 2). Within each row and temperature, means followed by different superscript letters indicate significant differences (*p* < 0.05) according to Tukey’s B multiple-comparison test.

**Table 4:** Composition of protein–starch formulations prepared at a constant total solids content (17% *w*/*w*).

Starch Substitution Level (%)	Protein (g)	Starch (g)	Total Solids (g)
0	4.25	0.00	4.25
10	3.83	0.43	4.25
15	3.61	0.64	4.25
20	3.40	0.85	4.25
25	3.19	1.06	4.25

## Data Availability

The raw data supporting the conclusions of this article will be made available by the authors on request.
